# Translucent poly(vinyl alcohol) cryogel dosimeters for simultaneous dose buildup and monitoring during chest wall radiation therapy

**DOI:** 10.1120/jacmp.v17i5.6148

**Published:** 2016-09-08

**Authors:** Molham M. Eyadeh, Mark A. Weston, Janos Juhasz, Kevin R. Diamond

**Affiliations:** ^1^ Physics Department Faculty of Science, Yarmouk University Irbid Jordan; ^2^ Juravinski Cancer Centre Hamilton ON Canada; ^3^ Department of Medical Physics and Applied Radiation Sciences McMaster University Hamilton ON Canada

**Keywords:** radiation therapy, build up, cryogel, breathing irregularities, chest wall, DIBH

## Abstract

Chest wall radiation therapy treatment delivery was monitored using a 5 mm thick radiochromic poly(vinyl alcohol) cryogel that also provided buildup material. The cryogels were used to detect positioning errors and measure the impact of shifts for a chest wall treatment that was delivered to a RANDO phantom. The phantom was shifted by ±2,±3, and ±5 mm from the planned position in the anterior/posterior (A/P) direction; these shifts represent setup errors and the uncertainty associated with lung filling during breath‐hold. The two‐dimensional absolute dose distributions measured in the cryogel at the planned position were compared with the distributions at all shifts from this position using gamma analysis (3%/3 mm, 10% threshold). For shifts of ±2,±3, and ±5 mm the passing rates ranged from 94.3% to 95.6%, 74.0% to 78.8%, and 17.5% to 22.5%, respectively. These results are consistent with the same gamma analysis performed on dose planes calculated in the middle of the cryogel and on the phantom surface using our treatment planning system, which ranged from 94.3% to 95.0%, 76.8% to 77.9%, and 23.5% to 24.3%, respectively. The Pinnacle dose planes were then scaled empirically and compared to the cryogel measurements. Using the same gamma metric, the pass rates ranged from 97.0% to 98.4%. The results of this study suggest that cryogels may be used as both a buildup material and to evaluate errors in chest wall treatment positioning during deep‐inspiration breath‐hold delivery. The cryogels are sensitive to A/P chest wall shifts of less than 3 mm, which potentially allows for the detection of clinically relevant errors.

PACS number(s): 87.55.km, 87.57.uq

## I. INTRODUCTION

The organs of the cardiopulmonary system are at risk during radiation therapy of the thorax. Previous studies have shown a strong correlation between the irradiated cardiac volume with cardiac mortality,^(1)^ and the irradiated lung volume associated with functional lung damage.^(2)^ Meta analyses have shown cardiac deaths following breast radiotherapy are associated with the volume of the heart receiving dose in excess of 5 Gy.^(3)^ Cardiac toxicity may be exacerbated during radiation therapy to the left breast or chest wall.^(4)^ Thus, there is a desire to balance the dose delivered to the clinical target volume (CTV) and dose to the healthy organs at risk (OAR).^(5)^

Patients receiving mastectomies often receive adjuvant radiotherapy to reduce their risk of local recurrence and in turn this improves long‐term survival.^(6)^ Radiation therapy of the chest wall is typically delivered by using photon beams and three‐dimensional conformal radiation radiotherapy or forward‐planned intensity‐modulated radiation therapy (IMRT).^(7)^

The dose delivered by these techniques may be affected by respiratory motion of the thorax during treatment.^8^, ^9^ These movements may be accounted for using breathing adapted radiotherapy (BART) techniques such as respiratory gating, breath‐hold (BH), and deep‐inspiration breath‐hold (DIBH).^5^, ^7^, ^8^, ^9^, ^10^ DIBH is a technique used in the treatment of left‐sided breast cancer that allows for cardiac sparing (i.e., increasing the distance between the chest wall target and heart). By instructing the patient to hold their breath during the treatment, the dose delivered to the cardiac and pulmonary volumes are reduced when compared to free‐breathing.^7^, ^9^, ^10^, ^11^ During DIBH, the patient takes a deep breath and holds it during CT simulation and subsequently during treatment. The treatment is delivered as long as the chest wall remains within a certain range of positions, which are tracked using an external fiducial on the thorax or abdomen. Different BH methods have been used, such as the spirometry‐based active breathing coordinator (ABC) system (Elekta AB, Stockholm, Sweden) and the video‐based real‐time position management (RPM) system (Varian Medical Systems, Palo Alto, CA). Both systems are viable options for delivery of a DIBH treatment.^7^, ^9^, ^11^ The dosimetric effect of DIBH depends on patient anatomy, lung capacity, and pulmonary function.^(9)^

The dose to the heart may also be reduced using techniques such as IMRT or volumetric‐modulated arc therapy (VMAT). The DIBH‐IMRT decreases the maximum dose to the heart for left‐sided patients compared with DIBH three‐dimensional conformal radiation radiotherapy, but the treatment time is often much longer.^(7)^ With VMAT, the target coverage is improved with substantial reduction in treatment time.^(6)^ Given the level of modulation in these types of treatment fields, all uncertainties and setup errors should be well understood in order to limit the potential risk of geographical misses leading to the underdosing of the target tissues and/or overdosing of healthy tissues.^(12)^ Positioning uncertainties and breathing motions can introduce a significant deviation between planned and delivered dose distribution.^(13)^ The typical maximum uncertainties/setup errors involved in left breast and chest wall treatment in the A/P direction (vertical chest wall excursion over the treatment duration) have been reported to be as large as 5 mm.^13^, ^14^, ^15^ Patient‐induced chest wall shifts of a similar magnitude during DIBH have also been reported.^7^, ^8^

A variety of dosimetric systems are used currently during radiotherapy for dose verification purposes, including thermoluminescent detectors (TLDs),^(16)^ metal‐oxide semiconductor field‐effect transistors (MOSFETs),^(17)^ and radiographic or radiochromic film.^(18)^ An alternative approach would be to use gel dosimeters.^19^, ^20^, ^21^ Dosimetric cryogels are flexible materials that conform easily to curved regions of the body. These cryogels may be used as both a buildup material and act as an *in vivo* dosimeter to monitor the treatment delivery.^(19)^ In regions where the surface dose is of interest, a dosimetric cryogel may provide an accurate estimation of superficial dose distributions. Cryogels may also be used to quantify uncertainties in setup or field conjunctioning, and breathing irregularities during left breast or chest wall DIBH radiation therapy.^20^, ^21^

Buildup materials are often used for chest wall radiation therapy to deliver the full prescribed dose up to the target tissue surface,^(16)^ where the typical thicknesses of buildup used for megavoltage photon beams range from 3–10 mm, depending on in‐house practices.^(22)^

The purpose of the work presented here is to use a translucent poly(vinyl alcohol) cryogel (PVA‐C) containing ferrous benzoic xylenol orange (FBX)^(23)^ to provide buildup and to monitor treatment delivery. The concept is demonstrated using radiation treatment fields delivered to the left chest wall region of a RANDO phantom (Phantom Laboratory, Salem, NY). To simulate setup errors in chest wall position during DIBH, irradiations were conducted at both the planned position and with small A/P shifts.

## II. MATERIALS AND METHODS

### A. Treatment planning system and dose delivery

**Figure 1:**
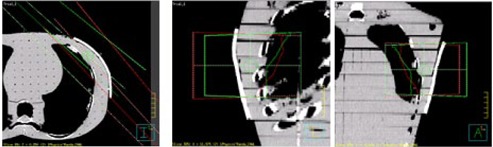
Treatment planning used in this study. Three plan views of the two tangential beam arrangements.

CT images of the RANDO phantom with cryogel placed over the chest area (5 mm thick piece $15\times 15\;{\text{cm}}^{2}$) were acquired with a 3 mm slice thickness (Brilliance Big Bore, Philips Radiation Oncology Systems, Fitchburg, WI). The CT dataset was exported to our radiation treatment planning system (Pinnacle 9.2, Philips Healthcare, Andover, MA) to generate a forward‐planned IMRT tangential chest wall treatment plan using $10\times 10\;{\text{cm}}^{2}$ 6 MV photon beams, at angles of 140°/314°, with no collimator or couch rotation segmented fields. Dose evaluation surfaces were defined in Pinnacle at the chest wall surface and in the center of the cryogel as part of a planning study; the procedure is described below. A jaw‐defined $8\times 12\;{\text{mm}}^{2}$ 6 MV reference beam was included in the plan outside of the irradiated region to act as a registration point. A prescribed dose of 1,000 cGy was planned for delivery to the surface of the chest wall. Figure 1 shows the field arrangement used in this study projected onto a three‐field view of the RANDO phantom.

The treatment was delivered using a Varian TrueBeam STx linear accelerator and the A/P shifts were performed using the PerfectPitch six‐degrees‐of‐freedom couch, which has a resolution of 0.1 mm (Varian). In addition to delivering the treatment at the correct position, the couch height was adjusted so that the phantom was shifted A/P by ±2 mm,±3 mm, and ±5 mm to simulate errors in chest wall position during treatment.

### B. Planar dose calculation using dose evaluation surfaces

Two‐dimensional dose maps were generated using Pinnacle's planar dose calculation tool. The dose evaluation surfaces were derived in a semiautomated manner. First, a contour of the radiochromic cryogel was generated by manual segmentation. Then, the external contour of the physical body, in this case the RANDO phantom, was generated automatically and confined to a bounding region of interest corresponding to the radiochromic cryogel. A contour representing the mid‐thickness of the cryogel was generated by expanding the external contour by 2.5 mm (i.e., to the center of the 5 mm thick cryogel) and confining the expansion to the cryogel. The physical coordinates corresponding to the cryogel‐RANDO interface, as well as the center of the cryogel, were extracted from the region‐of‐interest file (*.roi) and converted to a file format that was readable by the planar dose calculation utility. These surfaces were used to record the dose delivered in the planned position and with the isocenter shifted by ±2,±3, and ±5 mm from the planned position in the A/P direction to simulate chest wall positioning errors during DIBH. This planning study was used to establish the minimum required performance of the cryogel dosimeter system.

### C. Radiochromic cryogel dosimeter preparation

The cryogel dosimeter was prepared from a mixture of PVA, water, dimethyl sulfoxide (DMSO), and radiochromic FBX. The mixing procedure has been presented elsewhere,^(23)^ and so is only summarized here. 15% PVA by weight was selected for its reasonable fabrication time, sturdiness, ease of handling, and sensitivity. All chemicals used in the formulation were obtained from Sigma Aldrich (St. Louis, MO).

The PVA (99+% hydrolyzed, MW 146000–188000) was dissolved in 25 mM sulfuric acid (95%–98%), water, and DMSO (≥99%) at 120°C (20 / 80 water / DMSO by weight). The hydrogel was cooled to 50°C, at which point a (10 mL) solution of 0.55 mM ferrous ammonium sulphate (ammonium iron (II) sulphate hexahydrate, 99%), 5 mM benzoic acid (≥99.5%), and 1 mM xylenol orange tetrasodium salt, all dissolved in 25 mM sulfuric acid (95%–98%), were added to the hydrogel. To make up the desired total volume (100 mL), a 25 mM sulfuric acid–water/DMSO solution was stirred in after the PVA had been dissolved fully. The hydrogel was then evacuated for 15 min to remove unwanted air bubbles. The hydrogel was poured into custom plastic moulds with dimensions of $15\times 15\;{\text{cm}}^{2}$ and 5 mm thickness. The samples were then subjected to three freeze‐thaw cycles with each cycle consisting of a period of freezing for 18 hrs at −80°C and 6 hrs thawing at room temperature. The resulting cryogel dosimeter is flexible and can conform to most parts of a patient's body.

### D. Measurement of cryogel dosimeter using a charge‐coupled device (CCD) camera apparatus

**Figure 2:**
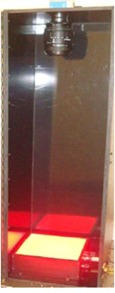
2D optical imaging apparatus consisting of a diffuse red light surface and a lens‐coupled CCD. Excess area on the light surface was masked using black construction paper to improve the dynamic range of the system.

Pre‐ and post irradiation scans of the cryogel dosimeter were acquired using the 2D imaging system presented in Fig. 2. The 2D imaging apparatus consists of a CCD camera (Nikon Corporation, Tokyo, Japan); a 28–105 mm, f/1.4–5.6, a (UC‐II) zoom lens (Sigma Corporation, Fukushima, Japan); and a Lumen‐Essence BK‐600 uniform red light‐emitting‐diode (LED) array (Luminus Devices Inc., Billerica, MA) housed in a light‐tight box. The lens was focused onto the center of the cryogel dosimeter to optimize the resolution. 2D images were collected by the 16‐bit CCD and stored as “TIFF gray image” files. Noise in the CCD images was reduced through post processing using the “wdencmp” denoising filter algorithm native to the MATLAB image‐processing toolbox (MathWorks Inc., Natick, MA). Pre‐ and post irradiation images were registered using a template that attached to the LED light source. 2D absorption coefficient maps were generated using an in‐house MATLAB program. The CCD camera had a resolution of 1,392×1,024 pixels. The pre‐ and post irradiation measurements were performed at ambient room temperature. The pre measurements were acquired 10 min before irradiation, and the post measurements were acquired 2 hrs after irradiation.

### E. Calibration of cryogel dosimeter and film

Translucent cryogel dosimeter samples were irradiated with a 6 MV photon beam at $20\times 20\;{\text{cm}}^{2}$ field size. The dose response was measured to establish the relationship between absorption coefficient and dose measured using the cryogel dosimeter. The calibration samples were 5 mm thick with a cross‐sectional area of $7\times 7\;{\text{cm}}^{2}$. Calibrations were performed at isocenter height (1 m source‐to‐axis distance) with the samples sandwiched between 5.6 cm slabs of polystyrene. Doses ranging from 100 to 4,000 cGy were applied with a dose rate of 633 cGy/min. The expected doses in the cryogel samples were computed with the treatment planning software.

The same process was performed to correlate the optical density of EBT‐2 Gafchromic film to dose (lot #A052810‐01) (International Specialty Products, Wayne, NJ). 6 MV photons and a $20\times 20\;{\text{cm}}^{2}$ field size were used. Doses ranging from 100 to 1,500 cGy were applied with a dose rate of 633 cGy/min. The expected doses in the film were also computed with the treatment planning software. As described later, film was used to validate cryogel measurements. The film was scanned using an Epson 11000XL Scanner (Proscan, Avision, Australia) and analyzed using Film QA Pro (Ashland Inc., Wayne, NJ). The red channel data were used at a resolution of 150 DPI. All films were read out approximately 24 hrs after their irradiation.

### F. Two‐dimensional dose analysis

Gamma analysis is used to judge the agreement between measured and calculated dose planes. Pixels that satisfy the evaluation criteria are assigned a value falling between 0 and 1; failing pixels are represented by values greater than 1.^(24)^ The passing rate represents the percentage of pixels passing compared to the total number of tested pixels (i.e., those pixels above the threshold dose).^(24)^ In order to compare Pinnacle dose planes directly with the cryogel measurements, two approximations are required. First, a scaling factor is necessary that accounts for dose computation deficiencies in the buildup region. Second, the distortion of the measured cryogel dose (flat) image must be ignored when comparing to the (curved) Pinnacle dose plane. In our study, gamma analysis was performed to compare the two‐dimensional absolute dose maps at the planned position (shift=0 mm) with doses at all shifts (±2,±3, and ±5 mm) for the radiochromic cryogels (measurement to measurement), and Pinnacle dose surfaces (calculation to calculation). The Pinnacle dose planes were computed using the skin surface and mid‐cryogel for all A/P shifts and were also compared to the measured cryogels after applying an empirical correction factor.

The gamma analysis was performed with the Film QA Pro analysis suite using 3%/3 mm criteria and a 10% threshold dose. All images were aligned using the dose resulting from the 6 MV $8\times 12\;{\text{mm}}^{2}$ jaw‐defined reference beam. The gamma analysis was restricted to the irradiated area excluding the reference beam. Three independent preparations and irradiations of cryogel at each shifted (A/P) position were performed. The reproducibility of the radiochromic cryogel manufacturing and treatment delivery was verified by intercomparing the triplicate irradiations performed for each shifted position by using the same gamma analysis that was used to compare the dose distributions at different A/P positions.

### G. Validation of cryogel dosimeter using film measurements

The PVA‐FBX‐C surface dose measurements were validated by comparing with film dose measurements. A $7\times 7\;{\text{cm}}^{2}$ piece of EBT‐2 film was placed under a 5 mm thick, $7\times 7\;{\text{cm}}^{2}$ cryogel dosimeter sample on the surface of a 10.4 cm thick slab of polystyrene. The cryogel/film stack was positioned at isocenter, with 100 cm SSD to the polystyrene surface. The system was irradiated at normal incidence using a $3\times 3\;{\text{cm}}^{2}$ 6 MV field. A total of 1,000 MU were delivered with a dose rate of 600 MU/min.

## III. RESULTS

The cryogel was calibrated under full scatter conditions using $7\times 7\times 0.5\;{\text{cm}}^{3}$ samples. The relationship between the measured absorption coefficient and expected dose in the linear range was $(3.00\pm 0.04)\times 10^{-4}\;{\text{mm}}^{-1}\;{\text{cGy}}^{-1}$, which is consistent with previous measurements.^(23)^ The calibration curves were used to convert attenuation maps measured with the film and cryogel into absolute dose.

Film QA Pro software was used to align and analyze the measured cryogel and stack film dose maps. The mean ratio between the cryogel and film for the irradiated area, computed point‐by‐point in the irradiated area was 0.749±0.005. This ratio can be estimated by integrating the percent depth‐dose (PDD) data (e.g., Varian Golden Beam data) over the gel thickness and dividing by the PDD at 5 mm (i.e., where the film is located). For the linear accelerator used in this study, the predicted dose ratio was 0.739, which agrees well with the measured value. The significance of this ratio comes from the fact the dose will not be uniform throughout the 5 mm cryogel material, and not equal to actual film measurement (representing surface dose measurement). However, by using this correction factor (i.e., by dividing the cyrogel dose by 0.749) the cryogel can be compared to the film.

Inaccuracies in the treatment planning beam model complicate direct comparisons between planned doses and dose measurements close to boundaries. However, it should be possible to compare computed doses on a defined surface, regardless of proximity to a boundary, for a variety of beam arrangements.

**Figure 3:**
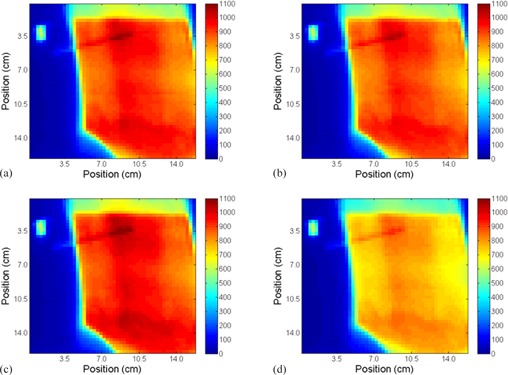
Absolute dose distributions maps in cGy calculated in Pinnacle at phantom‐cryogel interface at the different A/P positions from the planned position: (a) 0 mm, (b) +2 mm, (c) −3 mm, and (d) +5 mm.

The treatment planning system was used to generate 2D dose planes at the surface of the phantom and in the middle of the cryogel dosimeter. The absolute dose distributions were calculated at seven different A/P positions (0,±2 mm,±3 mm, and ±5 mm with respect to the planned treatment). The different A/P positions were achieved in Pinnacle by moving the beam isocenter by the shifted planned amounts. Figure 3 shows examples of the absolute dose distributions at the surface of the phantom at different A/P positions (0, +2, −3, and +5 mm).

The absolute dose planes from the shifted isocenters were compared to the same planes at the planned isocenter using gamma analysis. The passing rates ranged from 94.3% to 95.0%, 76.8% to 77.9%, and 23.5% to 24.3% at both the middle of cryogel and the surface of the phantom for shifts of ±2,±3, and ±5 mm. The results of the gamma analysis for both series of Pinnacle dose planes (phantoms surface and middle of cryogel) are summarized in Table 1. A 3 mm A/P shift resulted in a gamma pass rate below 80%, suggesting that an unacceptable fraction of the irradiated area at the chest wall surface may have been over‐ or underdosed.

**Figure 4:**
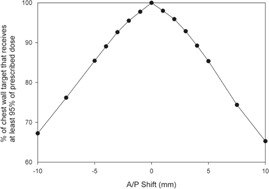
The fraction of chest wall target covered by the shifted 95% isodose levels as a function of A/P shift.

The clinical significance of the A/P shifts on the chest wall dose was examined using a simple planning study. The 95% isodose line arising from the planned position was converted into a volume representing the target tissue. The plan was delivered at the different A/P positions as described above, and the respective 95% isodose volumes compared to the planned “target.” The fraction of the target covered by the shifted 95% isodose levels is shown in Fig. 4 as a function of A/P shift. The data suggest that a 3 mm shift results in a reduction in coverage of approximately 7%, which may be considered clinically significant.

**Figure 5:**
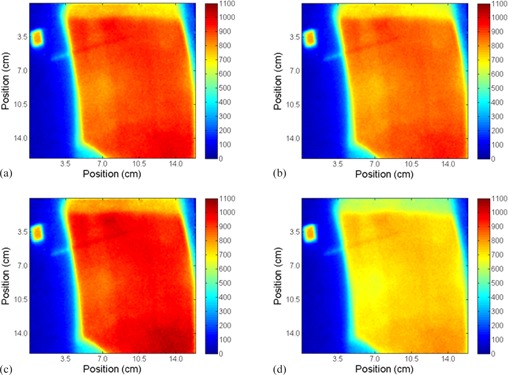
Absolute dose distributions maps in cGy measured using the cryogel dosimeter at the different A/P shifts from the planned position: (a) 0 mm, (b) +2 mm, (c) −3 mm, and (d) +5 mm.

**Figure 6:**
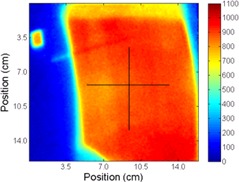
The 0 mm offset measured cryogel dose distribution map in cGy with black lines showing the crossline (horizontal) and inline (vertical) axes used to extract profiles at different A/P shifts, shown in Fig. 7.

**Figure 7:**
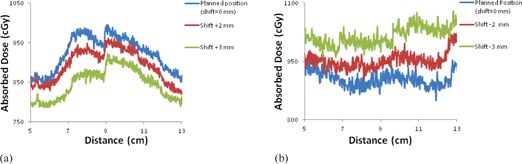
The crossline profiles (a), and inline profiles (b) extracted from absolute measured cryogel dose distribution images at different A/P shifts. The crossline profiles are taken from cryogels (+ A/P couch shifts) and the inline profiles (‐ A/P couch shifts).

The treatment was delivered to the RANDO phantom (with cryogel over the left chest wall) at the planned position and for all of the different A/P offsets (i.e., ±2,±3, and ±5 mm). For each different A/P position the treatment was repeated three times, each with an unirradiated cryogel dosimeter. Figure 5 shows examples of the absolute dose distributions measured at different A/P positions (0,+2,−3, and +5 mm). Figure 6 shows crossline and inline directions used to extract profiles for comparison, which are shown in Fig. 7. For the extracted profiles, the average dose difference from the planned treatment position was −2.8% and −7.8% for the

**Table 1:** Gamma analyses (3%/3 mm, 10% threshold) passing rates of absolute dose planes created in Pinnacle for all A/P shifts compared to the planned A/P position.

*Shift (mm)*	*Surface of Phantom (%)*	*Middle of Cryogel (%)*
−2	95.0	94.7
+2	94.6	94.3
−3	78.9	78.5
+3	77.5	77.1
−5	24.3	24.1
+5	23.8	23.5

+2and +3 mm shifts, respectively. Similarly, the average dose difference from the planned treatment position was +2.6% and +7.3% for the −2 and −3 mm shifts, respectively.

The measured absolute dose distributions at the planned treatment position were compared to those that were acquired at different A/P positions using gamma analysis. For each shift there are nine corresponding gamma analyses, where each measurement is compared to all three planned position measurements to demonstrate reproducibility. For shifts of ±2,±3, and ±5 mm the passing rates ranged from 94.3% to 95.6%, 74.0% to 78.8%, and 17.5% to 22.5%, respectively. A summary of these data is shown in Table 2. The reproducibility of the treatment geometry and gel fabrication methodology was also validated by performing gamma analyses between all the cryogels acquired at the same A/P position. By this measure, the setups were highly reproducible as the lowest passing rate for any treatment setup was 97.6%. A summary of these data is shown in Table 3.

In order to tie the dose maps measured using the cryogels to the planned dose distribution, a single correction factor was computed from the mean point‐by‐point ratio of the Pinnacle dose map to the measured cryogel dose maps for all shifts. The mean scaling factor between the two sets of data was 0.790±0.003. The scaling factor is required due to deficiencies in the buildup region observed for many treatment planning systems. While we have determined this empirically for the purpose of this study, it may be possible to model the scaling factor using a simpler geometry and incorporating any dependence on the angle of incidence. The correction factor model may be applicable to a wider range of applications where bolus may be used. Another uncertainty that arises in the computation of this correction factor is what planned dose plane should be used to compare against the measurement. This study employs the mid‐thickness plane; while a sensible choice, the reality is closer to an integral over the entire thickness of the cryogel. An integral may not be practical, or necessary, depending on the dose grid resolution. Another issue that arises is the comparison of an image recorded from a flat surface (2D cryogel) with a dose map computed on a curved surface (3D Pinnacle dose map). To perform the analysis correctly would necessitate a transformation matrix from the space of the cryogel, lying on a flat surface, to an irregular curved surface representing the shape of the RANDO chest wall in this example. However, if we except the limitations brought about by the distortion (which may be tolerable in this case, given the size of the cryogel and the radius of curvature of the chest wall), then a comparison can be made. The mid‐cryogel Pinnacle dose planes were scaled up by the 0.790±0.003 correction factor prior to comparing them to the measured cryogel dose maps. Gamma analysis demonstrated excellent agreement between the measured and planned dose distributions, with pass rates ranging from 97.0%–98.4%, as shown in Table 4. While the comparison between measurement and planned dose planes may be somewhat uncertain, it is a crucial step in order to ensure the accuracy of the patient treatment position during their first fraction. All subsequent fractions may be compared against the baseline measurement, which we have shown to be a sensitive measure of A/P positioning.

**Table 2:** Summary of gamma analyses (3%/3 mm, 10% threshold) passing rates comparing the measured absolute doses acquired at different A/P shifts to those acquired at the planned position. For each (A/P) shift a total of nine gamma analyses were performed.

*Shift (mm)*	*Mean ± Standard Deviation (%)*	*Minimum (%)*	*Maximum (%)*
−2	95.2±0.6	94.4	95.6
+2	95.0±0.7	94.3	95.2
−3	76.4±1.7	74.4	78.8
+3	75.6±1.0	74.0	76.7
−5	19.6±1.3	17.9	22.5
+5	18.9±0.9	17.5	20.2

**Table 3:** Summary of gamma analysis (3%/3 mm, 10% threshold) passing rates comparing two cryogel absolute doses measurements with one reference cryogel measurement at a specific A/P shift position.

*Shift (mm)*	*Mean ± Standard Error (%)*	*Minimum (%)*	*Maximum (%)*
0	98.9±0.3	98.8	99.1
−2	98.6±0.2	98.3	99.0
+2	98.8±0.2	98.5	99.1
−3	98.6±0.3	98.3	99.2
+3	98.8±0.3	98.5	99.3
−5	98.5±0.2	98.2	98.9
+5	98.5±0.4	97.6	98.7

**Table 4:** Gamma analyses (3%/3 mm, 10% threshold) passing rates comparing scaled mid cryogel Pinnacle dose planes by the 0.790±0.003 correction factor to the measured cryogel dose maps.

*Shift (mm)*	*Mean ± Standard Deviation (%)*
0	97.0±0.6
−2	97.3±0.7
+2	97.6±0.8
−3	98.4±0.7
+3	98.0±0.6
−5	97.5±0.8
+5	97.9±0.7

## IV. DISCUSSION

It is interesting to note that the gamma pass rates for A/P shifts are consistent between measurements using the radiochromic cryogel and the Pinnacle dose surfaces. Both studies suggest that a 3 mm A/P offset from the planned position may lead to an unacceptable difference in dose at the chest wall surface (i.e., gamma passing rate below 80%). An alternative way to consider these results is that the gamma analysis itself may provide a sensitive metric for detecting unacceptable A/P positioning errors. In this context, a gamma pass rate less than a prescribed amount would indicate an unacceptable variation in patient position. Existing studies suggest that setup errors in the A/P direction can range from approximately 2 to 5 mm.^14^, ^15^ Additional studies have shown that patients may shift their chest wall position during free breathing (FB), BH, and DIBH by 2.5 2.7 and 4.6 mm, respectively.^7^, ^8^, ^14^, ^15^ Similarly, uncertainty in the superior/inferior (S/I) direction may also be significant during DIBH delivery, where patient‐induced shifts may be as large as 6.6 mm.^(7)^ We have focused on A/P shifts for clarity, although S/I shifts can be modeled in a similar way. Our data suggest that the larger A/P variations associated with DIBH would be readily discerned. Thus, the cryogel could be used to verify patient positioning during treatment delivery and possibly identify clinically relevant errors.

In this study, although the cryogels do not measure the dose to the heart directly, they can be used to detect errors in chest wall position during the treatment. The estimation of the dose received to both the chest wall surface and the heart (although indirectly) can be made. For this study, the dose given to the cryogel was 1,000 cGy, which is higher than the 175 to 255 cGy per fraction used for actual treatments. Such high dose (i.e., 1,000 cGy) is not mandatory for the cryogel as long as the cryogel is sensitive to the low doses also.

This new tool may be used as an *in vivo* dosimetry, capable of measuring surface dose. It may also be used to estimate chest wall position during treatment delivery compared to the planned position using gamma analysis, perhaps allowing for the estimation of dose outside of the bolused region. In addition, it is often difficult to shape and place standard types of bolus without air gaps. PVA cryogels are flexible enough to provide skin contact without gaps due to their rubbery and adhesive nature.

Treatments of other locations on the body with different thicknesses of cryogel may yield different results and would have to be tested. However, for chest wall treatments with a typical bolus thickness the results of this paper should be valid.

## V. CONCLUSIONS

Radiochromic cryogels were used to evaluate potential dosimetric errors observed during tangential irradiation of the chest wall arising from A/P shifts. A comparison of absolute dose measured using the cryogels for planned and shifted positions (±2,±3, and ±5 mm) resulted in gamma passing rates that ranged from 94.3% to 95.6%, 74.0% to 78.8%, and 17.5% to 22.5%, respectively. Similar results were obtained in the associated planning study, where the gamma pass rates ranged from 94.3% to 95.0%, 76.8% to 77.9%, and 23.5% to 24.3%, respectively, in the middle of the cryogel as well as at the surface of the phantom. These results suggest that the radiochromic cryogel used in this study can detect errors in chest wall position, and that an error as small as 3 mm in the A/P direction may represent a clinically relevant over‐ or underdosing of the chest wall target. A 3 mm offset may arise from a number of sources, whether it is an error in patient setup, breathing irregularity, or because, for DIBH, the patient may have shifted their body position to mimic a deeper inspiration than they may have achieved during the planning phase. Gamma analysis of independently produced samples suggested that excellent reproducibility in cryogel formulation, phantom setup, and treatment delivery was achieved within the study, where the minimum observed pass rate was 97.6%. Comparisons between the measured and planned dose planes agreed well after incorporating an empirical scaling factor, with gamma pass rates ranging from 97.0%–98.4%, allowing the measurement to be tied to a planned dose distribution. Given an increased interest in DIBH for left chest wall irradiation and escalating complexity of the associated radiation fields (e.g., VMAT), an *in vivo* dosimetry tool that provides buildup material and records dose in two dimensions to monitor treatment delivery may be desirable. While radiochromic films satisfy the 2D side of the equation, they may be difficult to place in a predictable position on the skin surface under added buildup material, whereas we are proposing a unified system that could be incorporated into the planning process more readily.

## ACKNOWLEDGMENTS

This work has been supported by the NSERC Discovery Grant program and the Yarmouk University Physics Department.

## COPYRIGHT

This work is licensed under a Creative Commons Attribution 3.0 Unported License.

## Supporting information

Supplementary Material

Supplementary Material
