# Supplementary figures and images for: Translucent poly(vinyl alcohol) cryogel dosimeters for simultaneous dose buildup and monitoring during chest wall radiation therapy
Source: J Appl Clin Med Phys. 2016 Sep 8;17(5):308–19. doi: 10.1120/jacmp.v17i5.6148 (PMC5874105; doi:10.1120/jacmp.v17i5.6148)

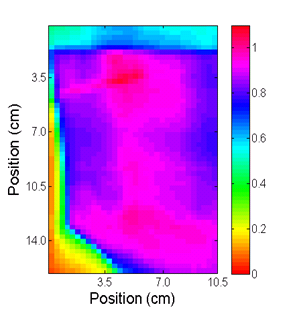

Supplement: Supplementary file 1 — Supplementary Material [file ACM2-17-308-s001.png]

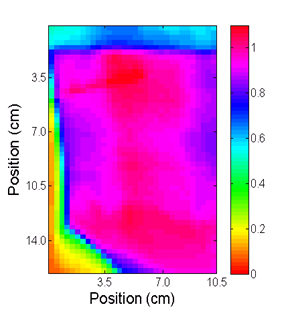

Supplement: Supplementary file 2 — Supplementary Material [file ACM2-17-308-s002.png]
